# Bromide of Ethyl, the Most Perfect Anæsthetic for Short, Painful Surgical Operations

**Published:** 1883-02

**Authors:** Julian J. Chisolm

**Affiliations:** Professor of Eye and Ear Diseases in the University of Maryland, Surgeon in Charge of the Presbyterian Eye and Ear Charity Hospital, Ophthalmic Surgeon to the University Hospital, etc.


AMERICAN JOURNAL

OF

DENTAL SCIENCE.

Vol. XVI. THIRD SERIES—FEBRUARY, 1883. No. 10.

### ARTICLE I.

### *Bromide of Ethyl, the Most Perfect Anæsthetic for Short, Painful Surgical Operations.*

BY JULIAN J. CHISOLM, M. D.,

Professor of Eye and Ear Diseases in the University of Maryland, Surgeon in Charge of the Presbyterian Eye and Ear Charity Hospital, Ophthalmic Surgeon to the University Hospital, etc.

(*Read before Balto. Academy of Medicine, Dec.* 5, 1882).

Three years since, when the Bromide of Ethyl was brought prominently forward as a substitute for chloroform by Dr. R. J. Levis and Dr. Laurence Turnbull, both of Philadelphia, I, with other surgeons, experimented with the new anæsthetic, with the intent of comparing its reputed advantages with the well-known agents sulphuric ether and chloroform. I discarded it after a very short trial on account of its apparent inefficiency, and because of the very evanescent nature of the sleep induced by it. I found great difficulty in puting my patients to sleep; and when at last narcotised, they would suddenly recover consciousness at most awkward periods in the midst of eye operations, to my serious annoyance. In two cases especially, in which I continued the inhalation from time to time as I would have done with chloroform; until upwards of an ounce of the bromide of ethyl was used, nausea and vomiting followed, which, in its severity and duration, I have rarely seen exceeded in the most sensitive of my chloroform patients. For twenty-four hours the sickness of stomach continued. The hospital ward in which the patients were lying had its atmosphere redolent with the garlicky odor of phosphorous and the breath of these patients was offensive from the same smell on the day after the inhalation. For some months after this very unsatisfactory brief experience my bromide of ethyl bottle remained corked. About this time great publicity was given to a death in the practice of Dr. Marion Sims, from ethyl administration, in which several ounces had been used, and the narcosis kept up for a long period. This was the first time that ethyl had been inhaled for anæsthetic purposes in New York City. This first fatal case was followed soon afterwards by a death under the use of ethyl in the practice of Dr. Levis, of Philadelphia These two fatal cases put a very sudden stop to the use of bromide of ethyl in the United States.

Confiding in the statements of Drs. Levis and Turnbull, both of them surgeons of large experience, that the bromide of ethyl had good properties, I was still disposed to beleive that the new and comparatively unknown anæsthetic possessed attributes which we had not succeeded in developing. I therefore again commenced experimenting cautiously with this new remedy. By degrees, as I became better acquainted with it, it secured my confidence. For the past year I have used it on an average at least once every day, often administering it four, five or six times, during the day's work in private practice and at the hospitals. Familiarities and effects, and the discovery of the proper method of administering it, has taught me to value its advantages more and more highly, till now I consider it *par excellence the anæsthetic* to be used for any painful surgical operation which can be quickly performed. *Having found out how to use it, and what to expect from its admin-*

*istration, I can obtain the most brilliant results from it, and have become quite enthusiastic in its praises.*

In every patient, using the needful precaution, I have produced complete narcosis in less than one minute, often in from twenty to thirty seconds. A deep sleep which, however, will not last more than one or two minutes. From this speedily induced narcosis recovery is rapid and complete, with neither nausea nor heaviness, so that as a rule five minutes after inhalation the patient is as much himself as if no anæsthetic had been used. Experience has taught me that these are the peculiarities of the bromide of ethyl when administered for anæsthetic purposes, and that as such they will prove of inestimable value to surgery.

The following very interesting cases, patients recently operated upon, will illustrate how thoroughly and speedily the brain resumes its full function after complete ethyl narcosis:

Miss M., a self-possessed little girl, eight years of age, desired to have an ugly squint corrected, and exhibited no timidity in witnessing the preparations needful for its performance. Prior to getting upon the table she had her collar loosened to remove any impediment to respiration. In doing so she took two roses from her dress and placed them on a vacant chair near by. She was then put on the operating table and the bromide of ethyl administered. A very few inspirations produced deep sleep, under which the tenotomy of the rectus muscle was performed. The ethylization and squint operation occupied fifty-six seconds. the time was taken by one of my assistants. Within three minutes from the commencement of the narcotism the child was perfectly awake, and was ready to get up from the table. When on the floor she walked at once to the chair and within four minutes from the time that the anæsthesia was commenced she was engaged in pining these roses into the front of her dress, with a composure which showed not only no present discomfort but a complete oblivion of the experience through which she had just passed.

*The second case*, also one of convergent squint, was that of a boy, fifteen years of age, who seemed very anxious to get rid of his deformity. After getting on the operating table, before the medical class at the University of Maryland clinic, I told him that when the towel was placed over his face it would have a very choking sensation, but that he could not choke from it. I also showed him how to take quick and full inspirations, so that the suffocative sensation would entirely pass away before he had breathed a half dozen times. When the folded towel, upon which a drachm of ethyl had been poured, was placed over his face, he commenced a most active respiratory movement, which in a very few seconds quieted down into a deep sleep. Within thirty seconds from the commencement of the ethylization narcotism was profound. The operation was commenced without delay and the division of the tendon speedily consummated. The entire operation from the commencing of ethylization to the perfection of tenotomy, did not exceed sixty seconds. A minute had not elapsed from the completion of the operation when he awoke, and jumping from the table to the floor of the amphitheatre he cried out in a jubilant voice, "I am all right," much to the amusement of the medical class who crowded the benches: a very different behaviour from that which follows the inhalation of chloroform or ether. In this case the entire period from the beginning of the inhalation, through the stage of complete narcosis, to perfect restoration, did not exceed two minutes.

A *third case* was that of a gentleman of extremely nervous temperament, was disfigured by a tarsal tumor. On account of his dread of being operated upon, he had carried this ugly swelling on his lid for over a year. One Sunday morning he presented himself at my office with the request that I would operate upon him at once, making as a condition that I would give him chloroform. It is an established rule with me never to administer an anæsthetic without an assistant being present. Having explained the necessity for this course, I requested him to meet me at the University Hospital within an hour, so that I could secure the presence and aid of the resident physician as my assistant. I anticipated his arrival and had everything prepared for his coming. I received him on his entering the vestibule of the hospital and accompanied him at once to the amphitheatre. With no loss of time he got upon the table and was told to take full inspirations of the medicated vapor in spite of the suffocative feeling excited by the ethyl. As soon as the cone containing a drachm of bromide of ethyl was placed over his nose and mouth, he commenced a series of slow, deep inspirations, which terminated in full narcosis by the time the eighth inspiration was taken. His breathing was free, pulse strong, color of face bright, with appearance in every repect of ordinary, deep, natural sleep. Desmarres' ring forceps or clamp soon secured the lid, the tumor was freely opened from the conjunctival surface, and by means of a cutting spoon the epithelial lining of the cyst was speedily scraped off. A few rapid rotations of the spoon effected this very promptly. This manipulation was a matter of a very few seconds. The awaking was equally prompt. Within two minutes from the time he laid upon the table he was again standing on the floor. Upon being questioned he said that he was perfectly himself, and had felt nothing whatever of the operation. He asked whether all was over and when assured of it he put on his hat and walked out of the room. Within six minutes from the time of his arrival he was again passing out of the entrance door of the hospital into the street, having, during this very short period been ethylized, operated upon and resumed his natural condition of feeling.

I might go on enumerating case after case, until my entire experience with the wonderful efficacy of ethyl, in all cases of what I now call primary anæsthesia, was gone through with, covering at this time over 400 inhalations. These three cases, however will suffice to show how thoroughly the brain recovers its perfect function after the deep but very transient impression brought about through the inhalation of the vapor of this potent agent. Persons who, only three minutes before, had been in such deep sleep that they were insensible to pain, now walking out of the operating room with a firm tread and with a clear brain.

On account of its activity, efficiency, and the evanescent nature of its narcotic effects, the bromide of ethyl has become my favorite anæsthetic for all surgical cases, in which, by quick manipulation, I can perfect a painful operation in a short period.

Experience, by daily administration, has taught me this very valuable lesson, viz., that the Bromide of Ethyl is not an anæsthetic which can be advantageously repeated or its inhalation be continued for any length of time. This is one of the serious mistakes which we made in our early experiments and which induce me, through ignorance, to discard the new agent as unreliable.

*Its wonderful action is obtained during the first minute of its inhalation and what I have called its primary anæsthesia.*

In cases, in which from some interference with the rapidity of the manual of operative procedure this primary anæsthesia wears off, and a second, and even more numerous administrations have to be made to keep up the anæsthetic state until the operation can be completed, while the narcosis can at all times be reproduced, nausea is very apt to follow. By this frequent repetition of the inhalation, a mental depression is established, as from the continued use of chloroform or ether, which may last many hours.

Fortunately there are many surgical operations of a very painful nature which can be perfected within the short period of a primary ethyl narcosis. Abscesses can be lanced, cysts emptied, sinuses laid open, wounds probed, strictures incised, muscles divided, ingrowing nails removed, surfaces cauterized, examinations made necessitating painful manipulations, and even amputations may be performed. It must not be forgotton that prior to the discovery of anæsthetics, Mr. Liston urgèd the general adoption of flap amputations, because all painful cutting, including the sawing of bones, could be completed in so many seconds and did not require minutes at the hands of dextrous surgeons.

In Eye and Ear Surgery, in which I am now exclusively interested, the irritable eyes of children can be carefully and thoroughly examined, tumors can be removed from the lids, abscesses punctured, orbital sinuses explored, the lachrymal canals laid open, the nasal ducts probed, foreign bodies removed from the cornea, canthotomy practiced, crossed eyes straightened, the operation for artificial pupil perfected, ingrowing lashes destroyed by the cautery, needle operations for soft or capsular cataracts effected, and even optico-ciliary neurotomy completed. All such operations I perform now under a primary ethylization, if the patient exhibits any timidity or expresses a desire to be put to sleep. Cataract extractions, enucleations and many lid operations require more time for their safe performance than ethyl narcosis permits. If every preparation be made in advance, instruments arranged in the order in which they are to be used, and placed within easy reach, and if the surgeon is able to manipulate with dexterity, it can be readily seen that a very large part of the painful procedures of surgical practice might be made altogether painless by taking advantage of thé wonderful nature of ethyl narcosis.

In Eye Surgery I not only use ethyl daily, but if deprived of it would feel that I had lost one of my very best assistants.

What can be more satisfactory than the correction of that ugly deformity, squint, under the perfectly quieting influence of the bromide of ethyl, in less than one minute, to cover ethylization and the tenotomy? In fifty-two seconds, as measured by the stop-watch, I have ethylized the patient and completed the division of the faulty muscle, The patient, quite himself in two minutes more, finds the ugly deformity gone, and without the slightest knowledge, on his part, of how the wonderful transformation has been brought about. This was my first expeditious operation. In the presence of the large medical class of the University of Maryland I have repeatedly completed the entire operation for the correction of squint, including the whole time necessary for the administration of the anæsthetic, in less than sixty seconds, as measured by the stop-watch.

*To use the bromide of ethyl efficiently, one must have confidence in himself and also in the safety of the agent which he is administering.*

For long operations, or such as I desire to complete slowly, I prefer to administer chloroform, an anæsthetic with which I have had a long, extensive and uninterruptedly satisfactory experience. *Of over* 12,000 *patients, upon whom I have operated under the narcotic effects of chloroform, I have not lost one.* These patients cover organic disorders of heart, lungs, kidney or visceral disease, in persons of all ages, from the child only a few days old to my oldest chloroform administration, a very old man of ninety-six. Some were strong while others were very feeble. I never refuse the comforts of an anæthetic to any person upon whom I have to operate.

Chloroform has always served me so faithfully that I have never had any good reason for transferring my allegiance to sulphuric ether. I now and then use ether but only at long intervals. Should a patient express any positive objection to chloroform and desire that ether be administered in his case, I always carry out his wishes. When the selection of the anæsthetic is left to me, and it usually is, my preference is decidedly for chloroform. I use chloroform so freely that I buy it literally by the gallon or in seven-pound bottles, many of which I have emptied. Of sulphuric ether I still have a pound bottle, which has been in my possession already five years, with contents not yet consumed. I believe that sulphuric ether is as safe as chlorform, but not more so. I know it to be more disagreeable in its odor and much more unpleasant in its inhalation I

believe that either chloroform or ether, when carefully given in accordance with well-known laws, which should always be observed in the inhalation of anæsthetics, will with few very rare exceptions carry safety in its train. I also believe that if proper care be not taken, trouble may come to both patient and surgeon regardless of the agent selected. Some physicians have much more anxiety while using anæsthetics than others, not because they have a worse class of patients, but because they have never acquired the necessary confidence in the article they use, nor do they feel the necessity, under conviction, of always having and observing fixed rules for their guidance in the use of these powerful agents.

After an experience of thirty years of an active surgical practice, I still hold chloroform to be the best of anæsthetics for tedious operations, provided certain simple rules are adhered to in its administration. I can enumerate them in a very few words.

1. I always, *without a single exception*, give a strong drink of whiskey, from one to two ounces, to every adult to whom I intend to administer chloroform. This is done a few minutes before they get on the operating table. Because I never omit this fundamental law, and in advance sustain the heart against the depressing effect of the anæsthetic, in not one of my 12,000 cases have I ever had to use, in a single instance, a hypodermic of whiskey. It is already in the stomach should it be needed and can do no harm if not required.

2. Always loose the neck and chest clothing so as to have no impediment to respiration.

3. Only administer chloroform in the recumbent posture with body perfectly horizontal and head on a low pillow, this pillow to be removed as the anæsthesia progresses.

4. Give chloroform on a thin towel folded in conical form with open apex so that the vapor, before inhalation, will be freely diluted with atmospheric air. In holding this cone over the face of the patient at some little distance from the nose, place the fingers under the borders of the cone for the double purpose of allowing air to enter freely, and also to prevent the chloroform liquid on the towel from coming in contact with the skin of the patient's face, and thereby avoid its blistering effects.

5. Should loud snoring occur force up the chin. This manipulation, by straightening the air passages from the nose to the larynx, makes easy breathing. The forcible elevation of the chin is far better in every respect than pulling out the tongue. It is easier of application, more quickly done, requires no instruments and is much more efficient in removing the impediment to respiration.

By always following these five simple rules I have had, so far, both safety and comfort in the administration of chloroform.

Possibly one very strong reason, why I have been so successful in the administration of chloroform is, that as a specialist in eye surgery the inhaler must be removed from the nose before I commence the surgical manipulations. Besides, while operating, I have constantly in view both the color of the face and the respiration of the patient, which I consider even more important for the surgeon to observe than to feel the pulse. When surgeons are operating on distant parts of the body and cannot watch the work of the administrator of chloroform, accidents are most apt to happen.

In the inhalation of the bromide of ethyl all of these rules laid down for the establishing of chloroform narcosis are not necessary, and some of them cannot be followed out.

The recumbent posture I consider essential for the safe administration of any anæsthetic, whether it be chloroform, ether or ethyl, hence these agents are not safe remedies at the hands of dentists, who place their patients in a sitting posture. Preparatory to the inhalation of the bromide of ethyl I have not found it necessary to give whiskey. The only precaution I take is to loose the neck clothing and have the patient lie down with the head only slightly elevated.

*My experiments have taught me that the mode of administering the ethyl should differ totally from that used in giving chloroform.*

Instead of chloroform vapor freely diluted with atmospheric air, a saturated ethyl vapor must be inhaled, to the exclusion of atmospheric air, in order to obtain speedily and effectually narcosis.

In my early experiments with this new agent I had not yet discovered this fundamental principle, and hence did not obtain good results. I voted bromide of ethyl a failure, because in common with other experimenters, I was too timid, or rather I should say too ignorant of its peculiarities, to push the ethyl vapor in the concentrated form, which I have since found necessary to obtain good results. By my present method of administering it, I can obtain perfect ethylization in patients in from twenty to sixty seconds, and have no after consequences of nausea or dullness of feeling.

The best inhaler for the giving of the bromide of ethyl is a thick towel folded into the form of a small cone with closed apex. Between one of the folds of the towel I place a sheet of paper, which makes the cone nearly air tight. The base of the cone must be wide enough to enclose both mouth and nose. The soft material of which the inhaler is made enables the rim to be kept firmly in contact with the face, so as to exclude air from entering. I always instruct the patient how to make long inspirations, and inform him that he must do this, notwithstanding the fact that he will feel somewhat stifled. I also try to give him confidence by assuring him that a very few inspirations will put him to sleep. Usually I make him go through the process of strong respiratory movements in advance, so that he will know exactly how to proceed. Into this towel cone I pour about one drachm of the bromide of ethyl and immediately invert the inhaler over the nose and mouth of the patient, holding its edge down firmly over the face. There is no fear of creating asphyxia, as all air cannot be excluded, and the height of the cone makes a considerable air chamber into which the patient breathes.

Children usually struggle to escape from the apparatus. *The cone, however, must not be removed from the face for an instant until anœethesia is produced.* At first some patients will resist the breathing of the vapor, but there is no fear that they will not catch their breath in time. Should children cry, it only insures inspiratory efforts, which the more surely and quickly will bring about the introduction of the vapor into the lungs. As a rule, a dozen full inspirations are all that are needed to produce deep narcosis. I recognize this desirable condition by a stoppage of all struggling. I have had deep sleep brought on by the sixth inspiration, when complete relaxation ensues, with quiet breathing, and an absence of reflex irritation should the conjunctive be touched. The patient retains the usual healthy color of lips and cheeks as if in ordinary sleep, and the pulse becomes slower and stronger as the narcosis becomes profound. Thirty seconds, as a rule, is sufficient to bring about this desirable condition, and have the patient ready for operation.

I have not found this anæsthetic sleep last more than two or three minutes, often not so long.

Usually the patients awake suddenly and as completely as they would do from ordinary sleep. They are able to get down from the operating table without assistance and walk off without staggering, and with brain clear to answer correctly any question: in fact, quite themselves.

It took me some time to acquire such confidence in the safety of the remedy, as to apply it in the concentrated form needful to obtain its fullest benefits. To the uninitiated it looks like cruel work to keep the cone of a saturated ethylized vapor over the face of a struggling patient. *I am convinced, however, that in no other way can quick, complete and safe anaethesia be obtained by it.* Fortunately the struggling is very soon over, and quiet sleep speedily ensues.

My experience with the bromide of ethyl will now exceed 400 cases, of which upwards of 300 are within the past year. I am beginning to be familar with its administration and its effects. *I now know what is to be obtained by it, and what not to expect from it.* I give it without hesitation, in any case, *to*

avoid painful manipulation. I have used it as often as six times a day, and I administer it, on an average, certainly once every day. In the last week I have given it fifteen times. For office use I find it invaluable, on account of its promptness, efficiency, evanescent nature of the anæsthesia induced, the abscence of nausea, and the perfect comfort with which patients operated upon can leave my office within a few minutes after the eythlization. Its use in my every-day experience does not interfere with the routine of office practice, nor occupy more time than I give to an ordinary office consultation, a very important desideratum to those who have restless patients awaiting their turn in the reception room.

Those who will use it by a single inhalation, to produce a short, deep sleep, and not resort to a mal-administration of this very valuable, powerful agent for a continued anæsthesia, which it is incapable of sustaining in safety and in comfort, will become as enthusiastic as I am over its brilliant results. They will in time learn to consider it, as I do, the most perfect of anæsthetic agents for quick, painful surgical work. It can never take the place of chloroform or sulphuric ether, where any heavy operations are to be done. These well-known and tried anæsthetics must continue in favor for all tedious operations, and will be used in minor surgery by those who manipulate slowly and who do not have prompt, quick assistants. But when one can take advantage of a primary anæsthesia, from the first administration of the bromide of ethyl, and having made every preparation in advance, will manipulate quickly, the new anæsthetic leaves nothing to be desired.

I will repeat, "can anything be more brilliant in surgery than a successful operation for squint, where an ugly deformity of years standing is promptly, thoroughly, safely and surely removed in less then one minute of time—fifty-two seconds for ethylization and operation ?" This is the nearest approach to magic in the art of surgery.—*Reprint from Md. Med. Jour.*

